# Single‐Cell Sequencing Reveals Heterogeneity and Interactions Between Epithelial Cells and Fibroblasts in Post‐ESD Oesophageal Stricture

**DOI:** 10.1111/jcmm.70411

**Published:** 2025-02-05

**Authors:** Lulong Tao, Junjun Xia, Die Hu, Guoxin Zhang, Yaoyao Gong, Jin Yan

**Affiliations:** ^1^ Department of Gastroenterology The First Affiliated Hospital With Nanjing Medical University Nanjing China; ^2^ The First Clinical Medical College Nanjing Medical University Nanjing China

**Keywords:** epithelial cells, fibroblasts, intercellular communication, oesophageal stricture, scRNA‐seq

## Abstract

Oesophageal stricture, especially circumferential lesions, is a common complication of endoscopic submucosal dissection (ESD). However, the exact mechanisms underlying its development remain unclear. Consequently, understanding tissue microenvironment changes is crucial for identifying therapeutic targets. To address this, single‐cell RNA sequencing (scRNA‐seq) was performed on oesophageal stricture samples and normal controls. Alterations in cellular composition were observed, particularly in epithelial, endothelial, fibroblast and immune cells. A notable increase was observed in the number of differentiating suprabasal cell_2 (DFSC_2), which displayed pro‐keratinizing traits. Detailed investigations revealed augmentation in a subset of these cells, characterised by elevated FTH1 and ECM1 expression, indicating their role in epithelial remodelling. Furthermore, fibroblast heterogeneity was demonstrated, with significant activation of myofibroblasts within stricture tissues. MDK–NCL, CXCL5/6‐CXCR2, and TGFA–EGFR ligand–receptor pairs were enhanced in stricture tissues, mediating epithelial–stromal interactions. This study dissected the transcriptional landscape of postoperative oesophageal stricture tissues, providing valuable insights into stricture mechanisms and potential preventive strategies.

## 1 Introduction

Endoscopic submucosal dissection (ESD) is a minimally invasive method for complete resection of early esophageal lesions [1, 2]. Although ESD mitigates surgical trauma and improves long‐term prognosis, it also poses potential complications such as bleeding, perforation and postoperative esophageal stricture [3]. Studies report a 90% incidence of esophageal stricture when the mucosal defect covers more than three‐fourths of the esophageal circumference [4, 5, 6]. Current clinical strategies for managing ESD‐induced esophageal stricture include endoscopic balloon dilatation, stent placement and pharmacological interventions [7, 8]. Additionally, there is growing interest in the therapeutic application of biomedical materials obtained from polymers and natural tissues [9, 10, 11]. These materials facilitate the adhesion and proliferation of mucosal epithelial cells, thus promoting the re‐epithelialization of artificial ulcers [12]. To date, few studies have reported the molecular mechanisms underlying esophageal stricture. Zhang and Xu [13] found that miR‐223‐3p, miR‐142‐5p, miR‐21‐3p and miR‐218‐5p interact with their target genes *FOXO1*, *PAX6*, *PIK3CA* and *ADRB1* to promote esophageal stricture formation. Accordingly, a comprehensive understanding of the microenvironmental changes following ESD is imperative for developing effective and simplified interventions.

Single‐cell RNA sequencing (scRNA‐seq) plays a crucial role in elucidating the intricacies of disease onset and progression [14]. Compared with traditional RNA‐seq, scRNA‐seq captures cell‐level details, revealing diverse cell populations and rare cell types with a higher resolution. Although it has been used to explore the progression of some fibrotic diseases, such as pulmonary fibrosis [15, 16] and liver fibrosis [17, 18], cellular heterogeneity and regulatory changes during the development of esophageal stricture post‐ESD have not been systematically studied.

This study employed scRNA‐seq to investigate the esophageal microenvironment in normal and postoperative stricture tissues. We identified transcriptional heterogeneity in epithelial and fibroblast populations, highlighting a specific epithelial subpopulation enriched in stricture tissues. Additionally, we investigated the epithelial–stromal crosstalk that may contribute to esophageal stricture formation post‐ESD.

## 2 Materials and Methods

### 2.1 Human Oesophagus Samples Collection and Single‐Cell Isolation

Oesophageal samples from patients with oesophageal stricture and healthy control were obtained from The First Affiliated Hospital of Nanjing Medical University. For eligible patients, fresh oesophageal tissues were collected via gastroscopy and cryopreserved in sCelLive solution within 30 min. After rinsing with Hanks Balanced Salt Solution and mincing, tissues were treated with sCelLive Tissue Dissociation Solution using the Singleron PythoN System at 37°C for 15 min. The cell suspension was filtered through a 40‐μm sterile strainer, and red blood cells were lysed using GEXSCOPE lysis buffer (1:2 volume ratio) for 5–8 min. After centrifugation at 300 × *g* for 5 min at 4°C, cells were resuspended in phosphate‐buffered saline (PBS) and stained with trypan blue for viability assessment.

### 2.2 Library Preparation and Sequencing

Single‐cell suspensions (2 × 10^5^ cells/mL) in PBS (HyClone) were loaded onto a microwell chip using the Singleron Matrix Single‐Cell Processing System. Barcoding beads captured mRNA, which was reverse‐transcribed into cDNA for PCR amplification. Amplified cDNA was fragmented, ligated with sequencing adapters, and used to construct scRNA‐seq libraries with the GEXSCOPE Library Kit [19]. Libraries were diluted to 4 nM, pooled and sequenced on an Illumina NovaSeq 6000 platform with 150 bp paired‐end reads.

### 2.3 Raw Data Processing of scRNA‐Seq Data

Raw reads from scRNA‐seq were processed to generate gene expression matrices using the CeleScope (v1.9.0; https://github.com/singleron‐RD/CeleScope) pipeline. Briefly, raw reads were first processed with CeleScope to remove low‐quality reads, using Cutadapt v1.17 [20] to trim the poly A tail and adapter sequences. Cell barcodes and unique molecular identifiers (UMIs) were extracted. Subsequently, STAR v2.6.1a [21] was used to map the reads to the reference genome GRCh38 (Ensembl version 92 annotation). UMIs and gene counts of each cell were obtained using featureCounts v2.0.1 [22] and used to generate expression matrix files for downstream analysis.

### 2.4 Quality Control, Batch Correction, Data Integration, and Major Cell Type Annotation

Downstream analysis was conducted using Scanpy v1.8.1 [23] in Python 3.7. Each sample was filtered to exclude: (1) cells with fewer than 200 genes or in the top 2% for gene count; (2) cells in the top 2% for UMI count; (3) cells with more than 50% mitochondrial content; and (4) genes expressed in fewer than five cells. Doublets were identified based on canonical cell marker expression patterns, and clusters enriched with multiple markers were removed. Batch effects were corrected using Harmony [24], followed by gene expression normalisation and scaling. The top 2000 variable genes were selected for principal component analysis (PCA), and clustering was performed at a resolution of 1.2. Significant differentially expressed genes (DEGs) were defined as genes expressed in more than 10% of cells with an average log(fold change) > 0.25. Cell type annotation was performed based on canonical markers from the existing literature.

### 2.5 Sub‐Clustering of Epithelial and Fibroblast Cell Types

Epithelial cells and fibroblasts were isolated from the integrated dataset and preprocessed as described previously. Based on previous reports [25, 26], epithelial cells were reanalyzed and categorised into quiescent basal cells, proliferating basal cells and differentiating suprabasal cells. Subsequently, differentiating suprabasal cells were classified as two clusters based on different transcriptional profiles. Fibroblasts and DFSC_2 were further sub‐clustered for more detailed analysis and refined annotation using specific markers.

### 2.6 Differential Expression Analysis and Functional Enrichment Analysis

DEGs between normal and stricture samples in each subset were identified based on an absolute log(fold change) > 0.25 and an adjusted *p*‐value < 0.05. Up‐ and down‐regulated DEGs were then screened for gene set enrichment analysis (GSEA), Gene Ontology (GO) and Kyoto Encyclopedia of Genes and Genomes (KEGG) enrichment analyses, using a cut‐off adjusted *p*‐value of 0.05 for significant results.

### 2.7 Functional Gene Signature Scoring

The potential functions of the cell subpopulations were assessed by computing functional module scores using Seurat's ‘Aucell’ and ‘AddModuleScore’ functions. Epithelial cell subpopulations were analysed using gene sets from ‘Hallmarker of cancer’ and ‘GOBP_EXTRACELLULAR_MATRIX_CONSTITUENT_SECRETION’ (https://www.gsea‐msigdb.org/gsea/msigdb/) [27]. Furthermore, ‘GOBP_KERATINIZATION’ was used to evaluate the keratinization capacity of the DFSC_2 subpopulation.

### 2.8 Construction of Pseudotime Trajectory for Single‐Cell Analysis

The cell differentiation trajectory was constructed using Monocle2 [28], utilising highly variable genes for spatiotemporal ordering. Dimension reduction was performed with the ‘FindVariableFeatures’ function from ‘DDRTree’, and the trajectory was visualised with the ‘plot_cell_trajectory’ function. CytoTRACE [29] was employed to validate the onset of differentiation.

### 2.9 Transcription Factor Regulatory Network Analysis

The transcription factor network was constructed using pyscenic v0.11.0 [30], leveraging the scRNA expression matrix and transcription factors from AnimalTFDB. GRNBoost2 predicted a regulatory network based on coexpression, while CisTarget excluded indirect targets and identified transcription factor‐binding motifs. Aucell quantified regulon activity in each cell.

### 2.10 Analysis of Cellular Communication Networks

CellChat [31] was used to analyse potential cellular communication patterns among epithelial cells, fibroblasts and other cell types. Initially, it identified differentially expressed ligands and receptors in various populations. We then calculated the probability of each interaction using the law of mass action, performed a random permutation test and constructed a weighted directed graph to represent the communication network.

### 2.11 Establishment of the Oesophageal Stricture Rat Model

Wistar rats (300–500 g body weight, regardless of sex) were used to construct an oesophageal stricture model. After fasting for 12 h before surgery, 5% pentobarbital was intraperitoneally administered at a dose of 0.5 mL/kg for anaesthesia. A specially designed balloon catheter was then inserted, and 0.1 mL of 20% sodium hydroxide solution was slowly injected through the catheter. The balloon catheter prevented sodium hydroxide reflux and entry into the stomach, inducing stricture at a specific oesophageal site.

### 2.12 Immunofluorescence

Oesophageal tissue samples from rats with induced stricture (*n* = 3) and healthy control (*n* = 3) were fixed in 4% paraformaldehyde for 15 min at room temperature. After fixation, tissues were permeabilised with 0.1% Triton X‐100 for 10 min. Following permeabilisation, non‐specific binding was blocked using 5% normal goat serum for 1 h. Samples were then incubated overnight at 4°C with primary antibodies. After washing with PBS, tissues were incubated with Alexa Fluor 488‐conjugated anti‐rat IgG and Alexa Fluor 594‐conjugated anti‐rat IgG secondary antibodies for 1 h at room temperature in the dark. Subsequently, nuclei were stained with DAPI for 5 min. Finally, samples were mounted on coverslips and analysed under a fluorescence microscope (Olympus Optics).

### 2.13 qRT–PCR

Total RNA was extracted using the TRIzol reagent (#9109, Takara) and reverse‐transcribed into cDNA using the reverse transcription system (#Q341‐02, Vazyme) following the manufacturer's instructions. Quantitative real‐time PCR (qRT–PCR) was performed using SYBR Green PCR Mix (#11203ES08, Yeasen) and the designed primers on an A46215 Detection System. β‐Actin served as the housekeeping gene. The primer sequences are shown in Table S1.

### 2.14 Statistical Analysis

Statistical analyses were performed using R (version 4.3.0), Python (version 3.7), and GraphPad (version 9.0.0) software. Continuous variables were compared using the Wilcoxon rank‐sum test or the Student's *t*‐test, as appropriate. All tests were two‐sided, with a *p*‐value < 0.05 considered statistically significant.

## 3 Results

### 3.1 Cellular Landscape of Post‐ESD Stricture and Normal Oesophageal Tissues

**FIGURE 1:**
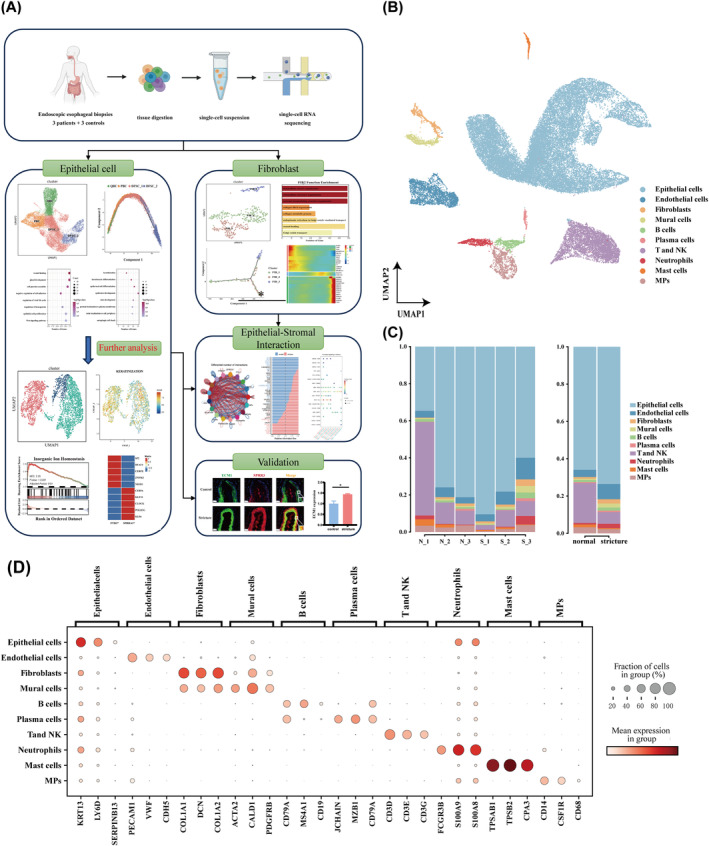
Overview of cellular heterogeneity in oesophageal tissues. (A) Primary design and workflow of the study. (B) Uniform Manifold Approximation and Projection (UMAP) of major cell populations in our study, with different coloured dots representing different cell types. (C) Percentage of various cell types in samples and between subgroups. (D) Violin plot showing specific marker genes for different cell subpopulations.

The flow chart of our study is depicted in Figure 1A. To understand cellular heterogeneity and mechanisms underlying oesophageal stricture after ESD, we collected samples derived from endoscopic biopsies (*n* = 3 from individuals with postoperative oesophageal stricture, and *n* = 3 from normal controls). After quality control and filtering, 40,906 cells were obtained (normal group: 16,502; stricture group: 24,404). Following preprocessing, sample integration and PCA, high‐quality cells were clustered into 23 clusters (Figure S1A–C). Based on well‐known marker genes (Figure 1D), we identified 10 cell lineages, including epithelial cells, endothelial cells (ECs), fibroblasts, mural cells, plasma cells, neutrophils, mast cells, T and NK cells, B cells and mononuclear phagocytes (MPs) (Figure 1B; Table S2). The proportions of each cell type varied across the six samples, with epithelial cells consistently compromising the majority. Compared with normal group, the stricture group displayed higher proportions of epithelial cells, ECs and neutrophils (Figure 1C), indicating a response involving tissue repair, neovascularization and inflammation post‐ESD.

### 3.2 Transcriptional Landscape of Oesophageal Epithelial Cells

Following ESD, disruption of epithelial integrity triggers the release of pro‐inflammatory cytokines, initiating an inflammatory cascade and early fibrotic events. During oesophageal stricture formation, early re‐epithelialization plays a crucial role in mitigating chronic inflammation and promoting tissue repair, serving as an indicator of treatment efficacy in clinical studies [32, 33]. Given that epithelial cells constituted the largest cellular component in oesophageal tissues (Figure 1C), we focused on their transcriptional properties during disease progression.

**FIGURE 2:**
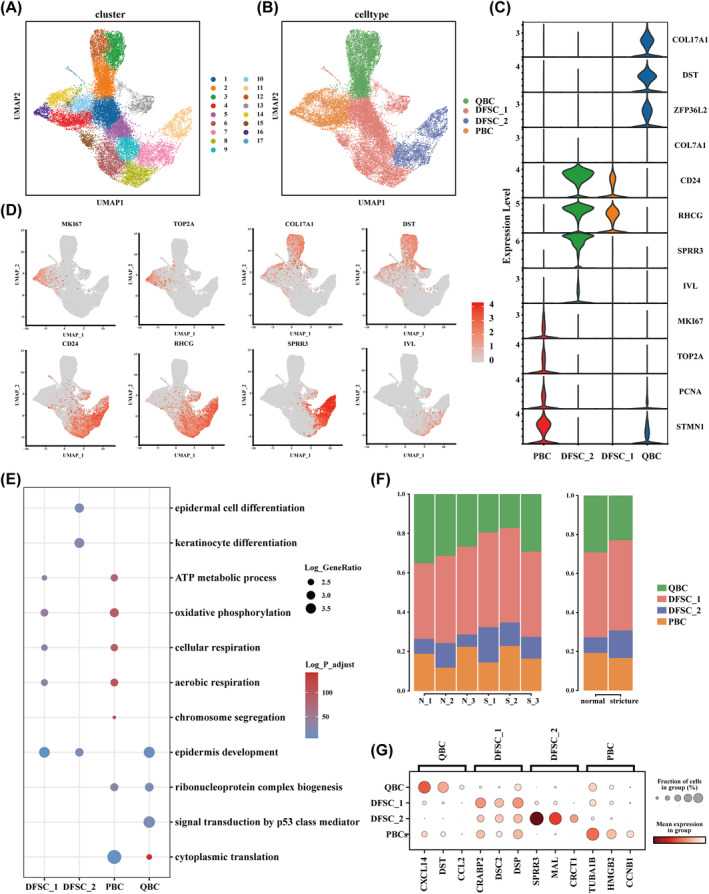
Reclassification and functional analysis of oesophageal epithelial cells. (A) Unsupervised clustering demonstrated 17 clusters of epithelial cells. (B) UMAP of further subdivided epithelial cells, colour‐coded by cell types. (C) Violin plot showing the specific marker of the four epithelial subclusters. (D) UMAP plots showing the expression and distribution of selected marker genes of each epithelial cell subcluster. Intensity of colour indicates average expression of genes. (E) Gene Ontology (GO) functional enrichment of different epithelial subclusters. (F) Stacked barplots showing proportions of epithelial cell subclusters across samples and between subgroups. (G) Dotplot showing the top three differentially expressed genes (DEGs) of each subclusters.

Unsupervised clustering initially identified 17 epithelial cell clusters (Figure 2A). Based on literature references [25, 26], we first assessed markers associated with proliferating and quiescent cell. Cluster 4, 10, 14 and 16 exhibited high expression of proliferative markers such as *MKI67* and TOP2A, and were classified as proliferating basal cell (PBC). Conversely, quiescent markers, including COL17A1, DST and ZFP36L2, were predominantly expressed in clusters 2, 3 and 12, leading to their designation as quiescent basal cell (QBC). For the remaining clusters, markers of differentiating suprabasal cell, including CD24, RHCG, SPRR3 and IVL were expressed in clusters 7 and 11, whereas other clusters expressed only CD24 and RHCG. Consequently, clusters 7 and 11 were labelled as differentiating suprabasal cell_2 (DFSC_2), wheresa the remaining clusters were defined as differentiating suprabasal cell_1 (DFSC_1) (Figure 2B). The expression profiles of markers for these four cell types are shown in Figure 2C,D. Additionally, the top DEGs are illustrated in Figure 2G and detailed in Table S3.

To further explore the functional roles of these epithelial subtypes, pathway enrichment analysis was conducted (Figure 2E). QBC, identified as multipotent stem cell, exhibited up‐regulated genes associated with protein synthesis, signal transduction and RNA processing, suggesting its adaptability to microenvironmental changes. PBC was enriched in pathways related to chromosome segregation and mitotic nuclear division, aligning with its proliferative capacity. DFSC_1 exhibited enhanced expression of genes involved in oxidative phosphorylation, aerobic respiration and ATP metabolism, highlighting its roles in tissue homeostasis. In contrast, DFSC_2 displayed elevated expression of keratinization‐related genes, including *SPRR3* and *CRCT1* (Figure 2G). GO biological process analysis confirmed the enrichment of pathways related to keratinocyte differentiation, epidermal cell differentiation, and skin development in this cell type (Figure 2E). Building on the functional characterisation of epithelial subtypes, we analysed their proportional changes in stricture tissues. As shown in Figure 2F, QBC and PBC exhibited a slight decrease, whereas both DFSC subtypes increased, with DFSC_2 showing a particularly marked rise.

### 3.3 Differential Genes and Pseudotime Trajectory Analysis in Post‐ESD Oesophageal Stricture Formation

**FIGURE 3:**
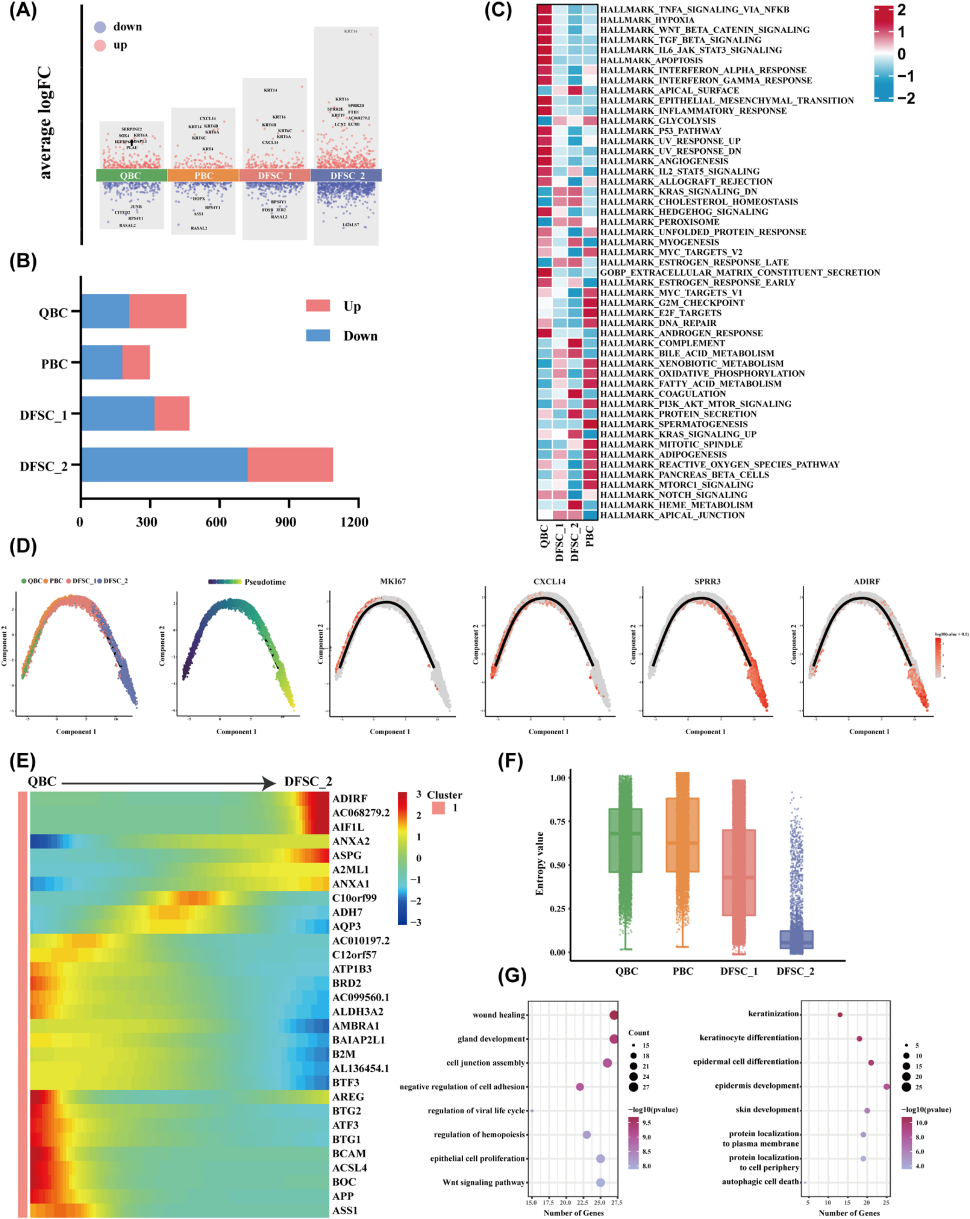
Differential and pseudotime trajectories analysis of epithelial cell subclusters. (A) DEGs of four epithelial cell subclusters between normal and stricture groups. Red represents up‐regulated genes, whereas blue represents down‐regulated genes. (B) Number of DEGs among the four subclusters between groups. (C) Heat map showing geneset score across four epithelial subpopulations. (D) Trajectory analysis via Monocle 2 of the epithelial cells, coloured by cell subclusters and selected gene expression across the pseudotime progression. (E) Heat map depicting DEGs (rows) across pseudotime (columns). (F) Boxplot showing the cytotrace score among QBCs, PBCs, DFSC_1 cells and DFSC_2 cells. (G) GO functional enrichment of the up‐regulated DEGs among QBCs and DFSC_2 cells between groups.

To pinpoint the critical epithelial subtypes driving oesophageal stricture formation, we conducted a differential expression analysis between normal and postoperative stricture tissues across the four identified epithelial subtypes. Among these subtypes, DFSC_2 exhibited the most significant transcriptional changes, with 369 up‐regulated and 721 down‐regulated genes (Figure 3A,B). Strikingly, keratins such as KRT14, KRT16, KRT6A and KRT6B were up‐regulated across all subtypes in stricture tissues, particularly in DFSC_2. Furthermore, CXCL14, a key regulator of cell proliferation and migration during injury repair [34], showed high expression in both PBC and DFSC_1 (Figure 3A).

Subsequently, we conducted gene set variation analysis to delve deeper into the molecular signatures (Figure 3C). QBC exhibited prominent activation in processes linked to epithelial‐mesenchymal transition, inflammatory response, extracellular matrix secretion and TGF‐β signalling. In contrast, DFSC_2 showed stronger activities in coagulation, protein secretion and the complement system, emphasising its role in wound healing and barrier function. Meanwhile, PBC was enriched in pathways related to mitotic spindle formation, oxidative phosphorylation and the G2/M checkpoint. Finally, NOTCH signalling, essential for cell differentiation and tissue homeostasis, was enriched in DFSC_1 (Figure 3C).

To delineate distinct cell states, Monocle2 was used to analyse epithelial cell subpopulations. In both normal and stricture tissues, a singular trajectory without bifurcations was observed, progressing from QBC to PBC, then DFSC_1, and finally DFSC_2 (Figure 3D,E). As depicted in Figure 3D, the proliferation marker MKi67 was predominantly expressed at the early stage, CXCL14 at the intermediate stage, and SPRR3 and ADRIF at the terminal stage. CytoTRACE analysis revealed that QBC and PBC had the highest scores, whereas DFSC_2 exhibited the lowest score, further supporting this trajectory (Figure 3F).

Combined with the altered cell ratios, we hypothesise that during mucosal re‐epithelialization, persistent inflammation disrupts normal epithelial proliferation and differentiation, driving excessive terminal differentiation. This shift likely results in an augment of keratinization function‐related DFSC_2, contributing to oesophageal stricture development.

We then explored disease‐induced functional changes in QBC (early differentiation) and DFSC_2 (late differentiation). QBC exhibited upregulation of genes related to wound healing and epithelial cell proliferation. Conversely, DFSC_2 displayed dysregulated expression of genes involved in keratinization and skin development (Figure 3G; Figure S2).

### 3.4 Identification of FTH1^hi^ DFSC_2 Epithelial Cells

**FIGURE 4:**
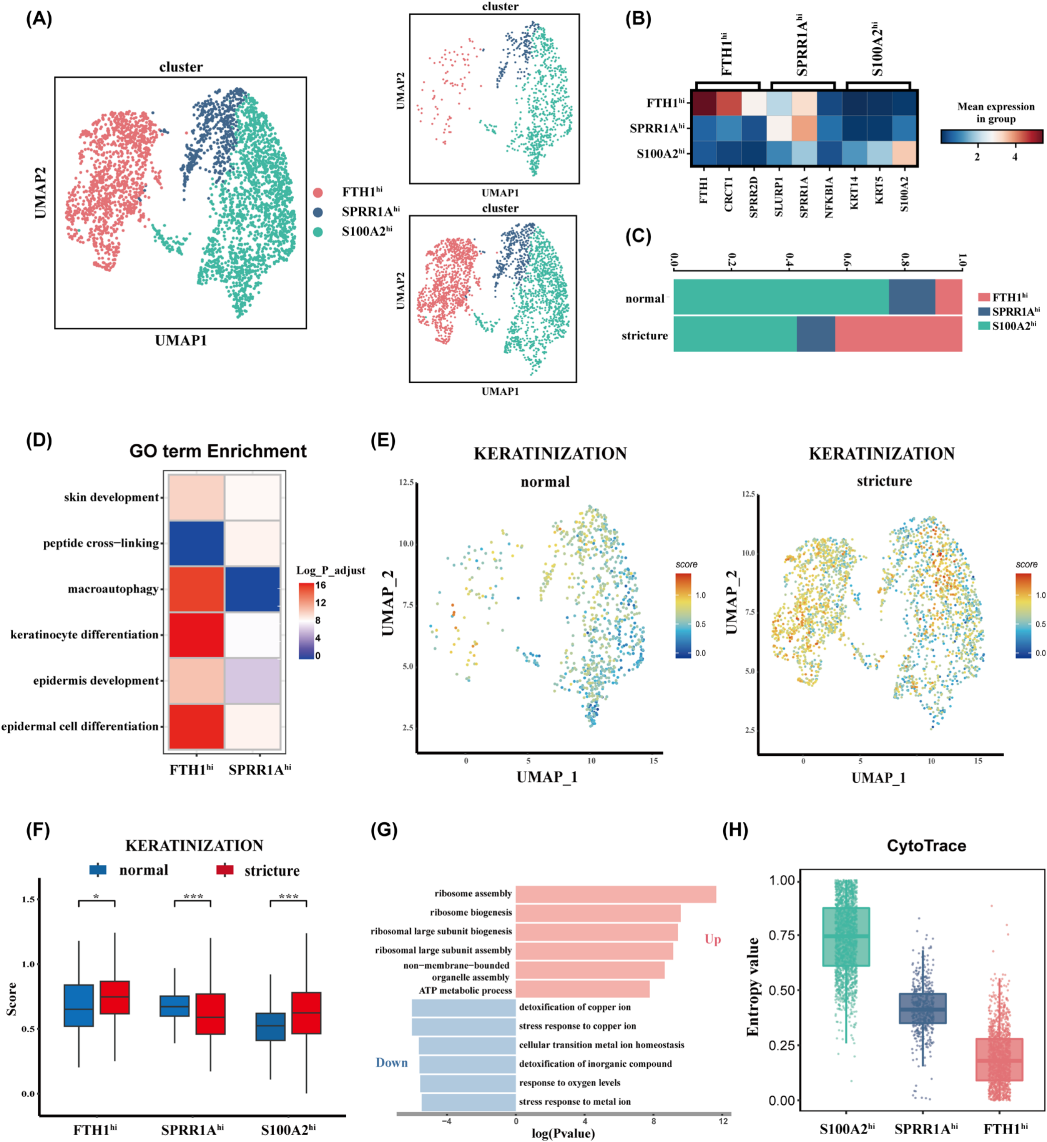
Identification of the novel FTH1^hi^ DFSC_2. (A) UMAP of the three states in DFSC_2. (B) Heat map showing the top three DEGs of each state. (C) Barplot showing the proportion changes of the three states. (D) Enrichment of GO terms for the FTH1^hi^ and SPRR1A^hi^ states. (E) UMAP plot showing the KERATIZATION score of each DFSC_2 cell subpopulation. Colour intensity indicates the score. (F) Boxplot showing the KERATIZATION score change of the three states between groups. (G) GO functional enrichment of DEGs in the FTH1^hi^ state between normal and stricture tissues. (H) Boxplot showing the cytotrace score among the FTH1^hi^, SPRR1A^hi^ and S100A2^hi^ states.

The findings suggested that DFSC_2 may be involved in oesophageal stricture formation. To investigate this further, we re‐clustered DFSC_2 into three distinct subclusters. Based on the expression patterns of the top DEGs, we defined these subclusters as FTH1^hi^, SPRR1A^hi^, and S100A2^hi^ DFSC_2 (Figure 4A,B; Figure S3; Table S4). Proportion analysis revealed an enrichment of the FTH1^hi^ DFSC_2 in the stricture group, whereas the S100A2^hi^ DFSC_2 was reduced (Figure 4C). Functional enrichment analysis indicated overlapping biological processes in the FTH1^hi^ and SPRR1A^hi^ DFSC_2, such as epidermis development and keratinocyte differentiation. However, FTH1^hi^‐specific genes were enriched in macroautophagy‐related pathways, while SPRR1A^hi^ DFSC_2 was linked to peptide cross‐linking. In contrast, the S100A2^hi^ DFSC_2 displayed distinct functions related to cytoplasmic translation and ATP metabolism (Figure 4D; Figure S4). Moreover, the Aucell algorithm showed increased keratinization activity in stricture tissues, with FTH1^hi^ DFSC_2 exhibiting the highest activity, further elevated during stricture formation (Figure 4E,F). Gene enrichment analysis demonstrated a suppression of pathways related to copper ion detoxification and metal ion homeostasis, alongside prominent upregulation of protein synthesis‐related genes in this subcluster (Figure 4G). Further trajectory analysis positioned the FTH1^hi^ and SPRR1A^hi^ subclusters in more differentiated states, suggesting a developmental progression from S100A2^hi^ to FTH1^hi^ DFSC_2 (Figure 4H).

**FIGURE 5:**
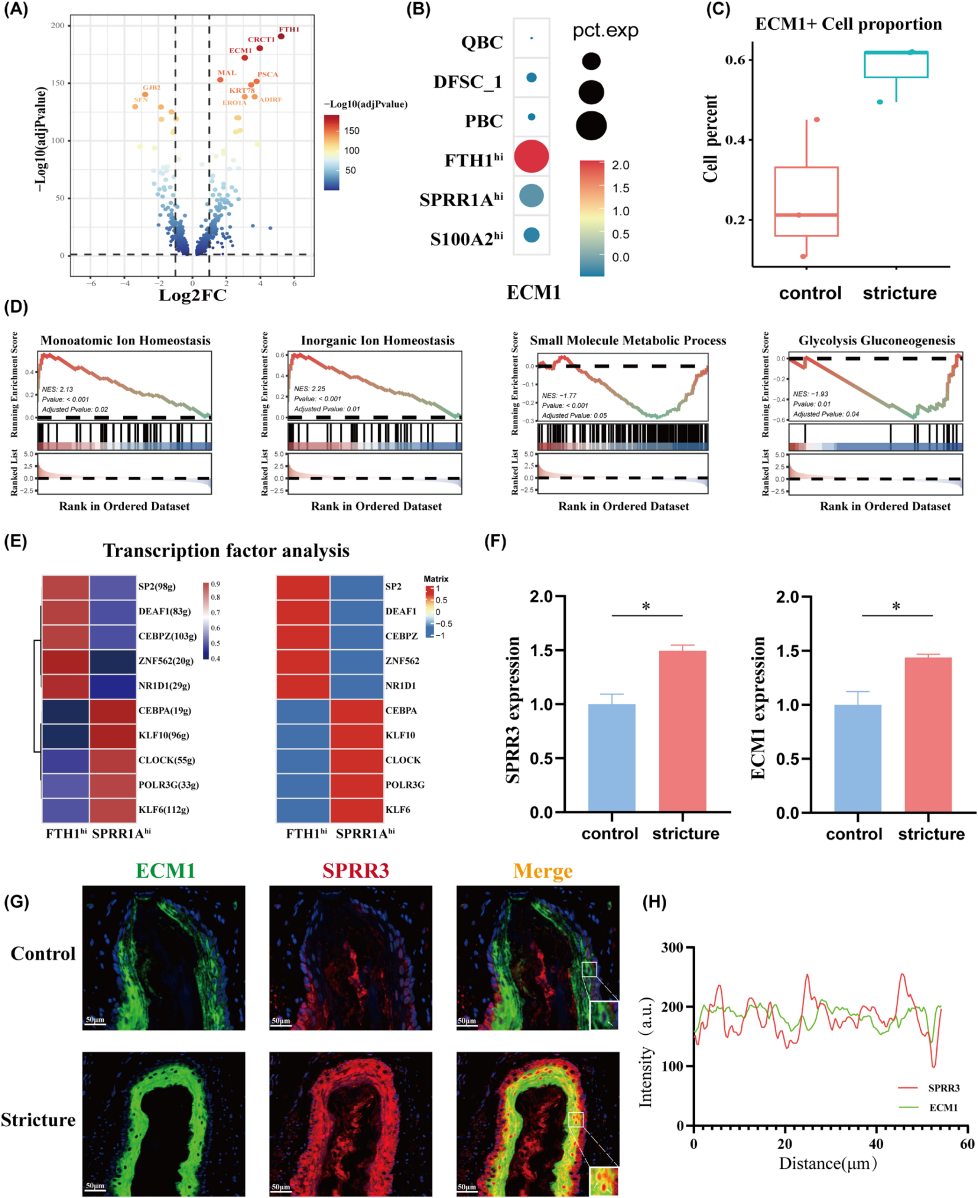
Distinct transcriptional features between the FTH1^hi^ and SPRR1A^hi^ states. (A) Volcano plot showing DEGs between the FTH1^hi^ and SLURP1^hi^ states, coloured by −log_10_(adj *p*‐value). (B) Dotplot of the average *ECM1* gene expression among different epithelial cell subclusters and boxplot of ECM1^+^ epithelial cell distribution between groups. (C) Boxplot illustrating the difference in the number of ECM1^+^ cells in normal and stricture tissues. (D) GSEA analysis for DEGs between the FTH1^hi^ and SPRR1A^hi^ states. (E) Heat map showing the area under the curve scores of average expression regulation by transcription factors, and average expression levels of representative transcription factor genes. (F) Histogram illustrating the fluorescence intensity of SPRR3 and ECM1 in normal and stricture tissues, *p < 0.05.(G) Immunofluorescent staining results showing the spatial distribution of ECM1 (green) and SPRR3 (red) in normal and stricture oesophageal tissues. Nuclei were counterstained with DAPI (blue). The merged image highlights areas of co‐localization (yellow). Scale bar = 50 μm. (H) Changes in fluorescence intensity and co‐localization of *SPRR3* and *ECM1* gene expression.

Given their functional similarity and advanced differentiation status, we further explored the heterogeneity of FTH1^hi^ and SPRR1A^hi^ DFSC_2. Transcriptional profiling revealed distinct gene expression patterns: FTH1^hi^ exhibited up‐regulated *ECM1* and *PSCA*, whereas SPRR1A^hi^ showed higher expression of *SFN* and *GJB2* (Figure 5A). Notably, epithelial cells expressing *ECM1* were significantly enriched in stricture tissues, particularly in the FTH1^hi^ DFSC_2 (Figure 5B,C). GSEA revealed that FTH1^hi^ DFSC_2 was enriched in pathways related to inorganic ion homeostasis and monoatomic ion homeostasis, while glycolysis and small molecule metabolism were enriched in SPRR1A^hi^ DFSC_2 (Figure 5D). To explore gene regulation heterogeneity, pyscenic analysis identified *ZNF562*, *NR1D1* and *SP2* as key transcription factors in the FTH1^hi^ DFSC_2, and *KLF10*, *KLF6* and *CEBPA* in the SPRR1A^hi^ DFSC_2 (Figure 5E).

Taken together, our analysis identified heterogeneity within the DFSC_2 subcluster, highlighting a tissue‐specific FTH1^hi^ epithelial subset with transcriptional alterations and enhanced keratinogenic activity. To validate these findings, we utilised tissues from a rat model of spontaneously induced oesophageal stricture. Quantitative fluorescence analysis showed significant upregulation of SPRR3 and ECM1 in stricture tissues, with evident co‐localization (Figure 5G,H; Figures S5 and S6A). These results confirmed the enrichment of the FTH1^hi^ epithelial cells identified via scRNA‐seq.

### 3.5 Different Subtypes of Fibroblasts Within Oesophageal Tissues

**FIGURE 6:**
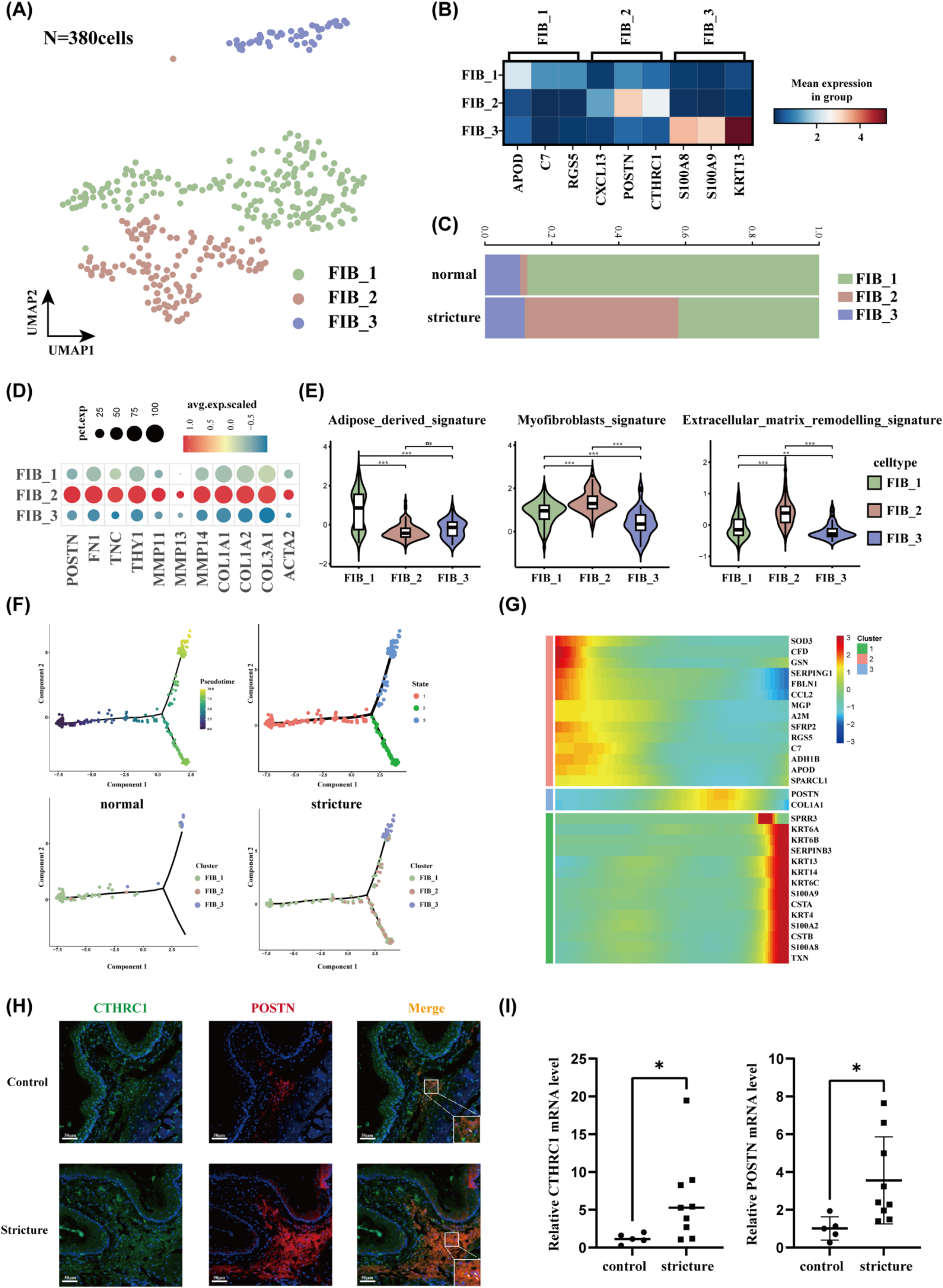
Heterogeneity of fibroblasts in oesophageal tissues. (A) UMAP of fibroblast subclusters. (B) Heat map showing the top three DEGs of each subcluster. (C) Barplot showing the proportion changes of the three subclusters. (D) Dotplot showing the average expression of the selected feature genes. (E) Violin plot of the gene signature scores for the three subclusters. (F) Trajectory analysis of the fibroblasts, coloured by cell subclusters. (G) Heat map depicting DEGs (rows) across pseudotime (columns). (H) Immunofluorescent staining results showing the spatial distribution of CTHRC1 (green) and POSTN (red) in normal and stricture oesophageal tissues. Nuclei were counterstained with DAPI (blue). The merged image highlights areas of co‐localization (yellow). Scale bar = 50 μm. (I) Quantitative RT‐PCR detection of mRNA expression of *CTHRC1* and *POSTN* in normal and stricture oesophageal tissues. *p < 0.05.

Fibroblasts are considered key drivers of oesophageal stricture, responsible for synthesising and depositing excess collagen, leading to scar formation and tissue remodelling [35, 36]. However, the disease‐mediated molecular changes and cellular heterogeneity remain poorly understood. Therefore, we conducted unsupervised clustering of fibroblasts and identified three subgroups: FIB_1, FIB_2 and FIB_3 (Figure 6A,B; Table S5). FIB_3 was equally distributed between the normal and stricture groups, whereas FIB_1 and FIB_2 showed significant shifts, with a marked increase of FIB_2 in the stricture group (Figure 6C). Our immunofluorescence results revealed a significant argumentation in CTHRC1 and POSTN double‐positive cells in the stricture tissues, accompanied by upregulation of corresponding mRNA levels (Figure 6H,I).

Next, we identified DEGs within each fibroblast subpopulation. In FIB_1, genes related to lipid metabolism and processing, including *APOD*, *A2M* and *ABCA8*, were highly expressed. This subpopulation also exhibited the highest adipose‐derived signature scores (Figure 6E). Interestingly, complement‐related genes such as *C3*, *C7*, *CFD* and *CFH* were also expressed in FIB_1 (Figure S7). GO and KEGG analyses revealed significant enrichment in blood coagulation, humoral immune response and complement and coagulation cascades, implying the complex biological functions of this subpopulation (Figure S8).

Genes encoding extracellular matrix (ECM) proteins, including periostin, fibronectin, tenascin C, Thy‐1, matrix metalloproteinases (MMPs) and collagens, were predominantly expressed in FIB_2 (Figure 6D). GO analysis indicated enrichment in terms such as ECM and collagen fibre organisation (Figure 6H). FIB_2 also showed the highest myofibroblast and ECM remodelling signature scores (Figure 6E), suggesting a strong myofibroblast inclination. In contrast, FIB_3 exhibited enrichment in genes related to cytoplasmic translation and epidermal development (Figure S8) while expressing epithelial markers KRT13 and KRT4, identifying it as EMT‐like fibroblast [37].

Subsequently, the differentiation sequence of these three subpopulations was investigated, revealing two trajectories, starting with FIB_1 as the initial stage and progressing to either FIB_2 or FIB_3 (Figure 6F). Notably, a transition from FIB_1 to FIB_2 was prominently observed in stricture tissues, suggesting its pivotal role in stricture formation (Figure 6F,G). Overall, these findings highlight the internal heterogeneity of fibroblasts and their microenvironmental changes during disease progression.

### 3.6 Cell–Cell Communication in the Tissue Microenvironment

**FIGURE 7:**
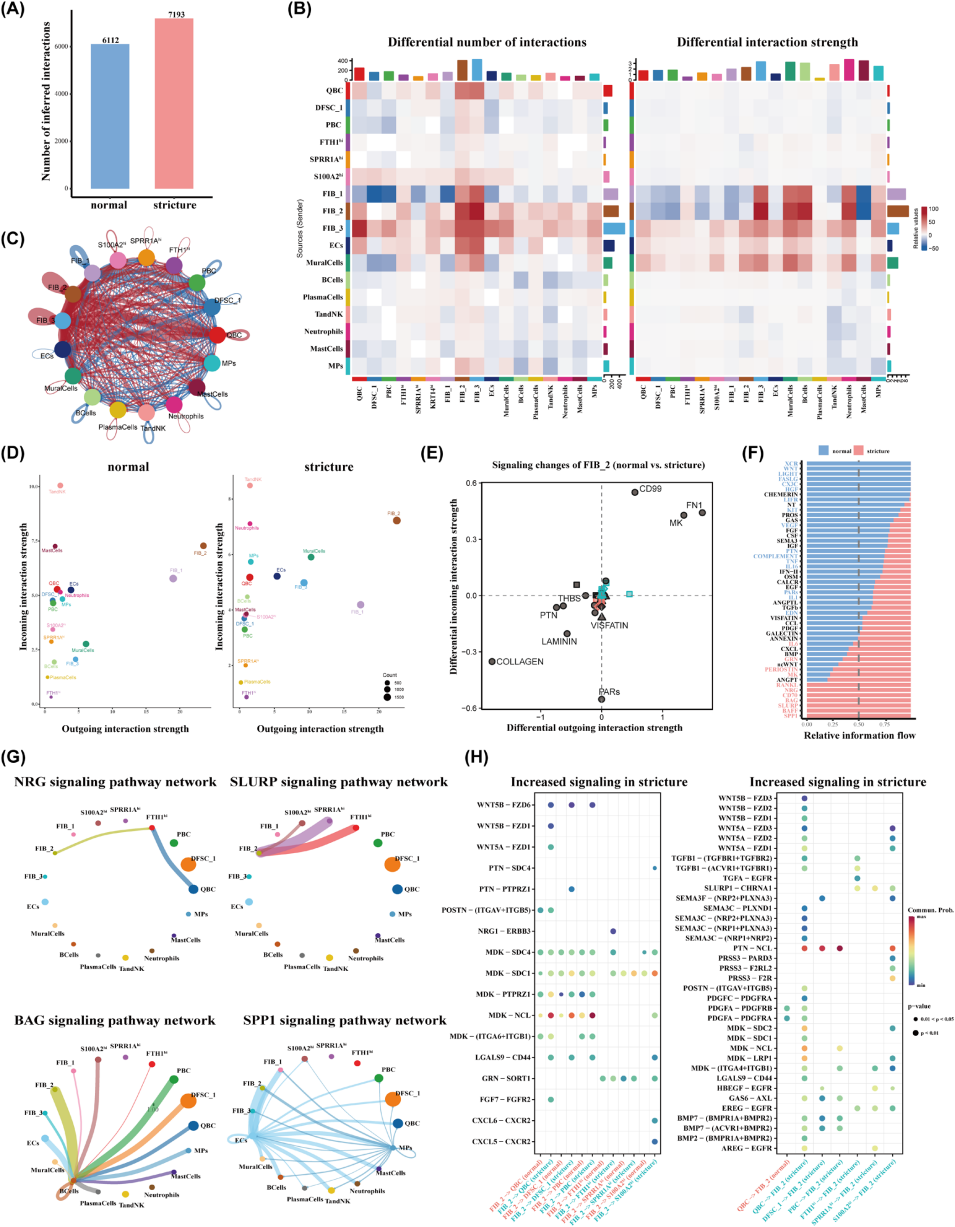
Overview of cell–cell communication in normal and stricture tissues. (A) Number of inferred interactions in normal and stricture tissues. (B) Heat map showing the differential number of interactions and differential interaction of strength of all cell types between normal and stricture tissues. (C) Circle plot showing the differential intercellular interactions between normal and stricture tissues. (D) Interaction analysis identifying prominently altered signalling sources and targets in normal and stricture tissues. (E) Altered signalling pathways that associate with FIB_2 cells between groups. (F) Information flow changes between stricture and normal groups based on the ‘Secreted signalling’ database. (G) Circle plot showing the specific signalling pathways up‐regulated in stricture tissues. (H) Up‐regulated ligand–receptor pairs between epithelial cells and FIB_2 fibroblasts.

Based on the above findings, differences in cell type proportions between groups likely reflect the complexity of intercellular communication. Supporting our hypothesis, CellChat analysis indicated the interaction strength was much greater in stricture tissues (Figure 7A). Moreover, fibroblasts in the stricture group showed increased interactions with QBC, ECs, MPs and neutrophils (Figure 7B,C). Further analysis of incoming and outgoing interaction strengths highlighted FIB_2 as a key player in the microenvironment across all conditions (Figure 7D). Comparison of specific signalling changes in FIB_2 showed significant activation of the FN1, MK and CD99 pathways in stricture tissues (Figure 7E).

Next, we compared changes in information flow using the ‘Secreted signalling’ database. The stricture group showed enrichment in several signalling pathways, including IL6, GRN, PERIOSTIN, MK, RANKL, NRG, CD70, BAG, SLURP, BAFF and SPP1 (Figure 7F). Compared with normal control, mural cells gained PERIOSTIN responsiveness, whereas QBCs, ECs and mural cells emerged as new signalling sources in stricture tissues (Figure S9). The SLURP signalling pathway mediated interactions from FIB_2 to all three states of DFSC_2 in the stricture group. Notably, the BAG pathway, originating from B cells, were received by all cell types except the SPRR1A^hi^ DFSC_2. In contrast, the SPP1 pathway, primarily sent by MPs and ECs, interacted with all cell types except FTH1^hi^ and SPRR1A^hi^ DFSC_2. The NRG pathway involved interactions from FIB_2 and QBC, with FTH1^hi^ DFSC_2 (Figure 7G).

Considering the pivotal role of FIB_2 as a hub for cellular communication, we analysed the differential ligand–receptor pairs between FIB_2 and epithelial cells. In the stricture group, MDK emerged as a potential ligand mediating the interaction between FIB_2 and epithelial cells, with SDC1 or NCL as major targeted receptors. Furthermore, the ligand–receptor pair NRG1‐ERBB3 was specifically enriched between FIB_2 and FTH1^hi^ DFSC_2. Interestingly, the inflammatory factors CXCL5 and CXCL6, secreted by FIB_2, were found to interact with S100A2^hi^ DFSC_2, suggesting their role in modulating epithelial inflammation and remodelling (Figure 7H).

Conversely, epithelial cells exhibited unique ligand–receptor interactions targeting FIB_2. In the stricture group, QBC showed the highest number of differential ligand–receptor pairs interacting with FIB_2. Among these, the PTN‐NCL axis predominantly contributed to the communication between epithelial cells (QBC, PBC, DFSC_1 and S100A2^hi^ DFSC_2) with FIB_2. Interestingly, TGFA, exclusively secreted by FTH1^hi^ DFSC_2, interacted with FIB_2 via the EGFR receptor, highlighting a distinct signalling pathway potentially involved in fibrotic remodelling (Figure 7H).

## 4 Discussion

In clinical practice, the incidence of oesophageal stricture post‐ESD has increased in recent years. This condition often induces dysphagia, necessitating multiple endoscopic dilatations [38]. Several studies have aimed to prevent stricture formation and promote wound healing by inhibiting inflammatory responses, promoting epithelial regeneration, or preventing intrinsic muscle injury [8, 11, 12, 39]. However, few of these studies have yielded effective results. To the best of our knowledge, our study presents the first comprehensive mapping of unbiased single‐cell landscapes in oesophageal stricture tissues. We identified a previously unreported subpopulation of differentiating suprabasal cells with high expression of FTH1, alongside pronounced fibroblast heterogeneity and myofibroblast activation in stricture tissues. Moreover, this research explored epithelial–stromal interactions in postoperative oesophageal stricture formation, uncovering potential therapeutic targets.

Histological analyses have confirmed that oesophageal stricture may arise from myofibroblast proliferation and activation, resulting in collagen secretion and ECM remodelling [40, 41]. For instance, elevated HIF‐1α expression in post‐ESD oesophageal tissues induces HK2 transcription and p38MAPK phosphorylation, accelerating fibroblast proliferation [42]. In our data, fibroblasts were not abundantly detected, likely due to the limited depth of endoscopic biopsy sampling. Nevertheless, we identified three distinct clusters: FIB_1, FIB_2 and FIB_3. Among these, FIB_2, originating from FIB_1, was notably enriched in stricture tissues. Additionally, this cell population exhibited molecular characteristics of myofibroblast, in line with the histological findings mentioned above.

Numerous studies have highlighted the crucial role of epithelial cells in oesophageal‐related disorders, such as their involvement in promoting the initiation and progression of oesophageal cancer [43]. Our study identified four epithelial cell types, including QBC, PBC, DFSC_1 and DFSC_2, which follow a differentiation trajectory in this sequence. Unlike the decrease in terminally differentiated cells reported in studies on eosinophilic esophagitis [26], our findings indicated a proportional increase in the number of terminally differentiated cells within stricture tissues. It has been reported that the repair process following ESD is initially marked by an inflammatory response, during which epithelial cell differentiation is intricately controlled by signalling pathways such as Wnt/β‐catenin and Notch [33]. The inflammatory infiltration within the local tissue microenvironment may disrupt these pathways, leading to pathological changes [44]. Based on this, we hypothesise that the inflammatory environment in postoperative artificial ulcers may interfere with the normal differentiation programme, leading to excessive differentiation activation and a marked accumulation of DFSC_2 in stricture tissues.

Functional analysis indicated that DFSC_2 was associated with keratinization and skin development. It is noteworthy that oesophageal mucosa is typically described as non‐keratinized [45, 46], despite the presence of a subset of keratin‐forming cells in normal epithelium that play vital physiological roles. Inflammation or trauma‐induced changes in epithelial state can disrupt cell balance, resulting in conditions like hyperkeratosis. Excessive keratin accumulation forms a stratum corneum, stiffening the mucosa, as seen in cases where oesophageal hyperkeratosis caused recurrent dysphagia [47]. Taken together, we conclude that epithelial keratinization contributes to oesophageal stricture formation. Interestingly, we identified a novel DFSC_2 subpopulation characterised by high FTH1 expression, significantly enriched in stricture tissues. In a previous study [48], FTH1 was found to play a key role in iron ion storage and release, and its upregulation has been reported in fibrotic renal tissues. Additionally, changes in the labile iron pool in macrophages were found to be associated with alterations in cellular functions, metabolism and oxidative stress capacity. Our study found that detoxification processes, copper ion stress responses and oxygen‐related biological processes were down‐regulated in FTH1^hi^ DFSC_2 within stricture tissues. We propose that FTH1^hi^ DFSC_2 exhibits a weakened cellular ability to counteract oxidative stress after surgical trauma, potentially leading to altered functions such as increased keratin synthesis. Moreover, this subpopulation displayed elevated *ECM1* gene expression, a critical regulator of ECM remodelling. However, ECM1 exhibits functional heterogeneity in various cell types and tissues. During myocardial infarction, ECM1 produced by macrophages and pericytes/vascular cells, binds to the fibroblast surface receptor LRP1, promoting fibrosis [49]. Conversely, in the liver, ECM1 knockout induces spontaneous hepatic fibrosis [50]. In summary, our findings suggest that targeting ECM1 in FTH1^hi^ DFSC_2 could be a potential therapeutic strategy.

Epithelial–stromal interactions are implicated in lamina propria remodelling and stricture formation, yet research in this area is limited. Unexpectedly, cell–cell communication analysis revealed stronger interactions between FIB_2 and upstream epithelial subpopulations than with DFSC_2. Specifically, FIB_2 secreted the ligand MDK, targeting the NCL receptor expressed on QBC, PBC and DFSC_1. MDK, a heparin‐binding growth factor, regulates crucial processes such as cell proliferation and angiogenesis in various cancers [51, 52]. In contrast, NCL, a cytosolic protein, is essential for transcription and RNA processing [53, 54]. The upregulation of MDK–NCL may drive upstream cell differentiation, underscoring its crucial role in oesophageal pathological remodelling.

Cellular senescence commonly occurs during wound healing, with senescent cells secreting a senescence‐associated secretory phenotype (SASP) comprising various inflammatory factors [55]. Senescent fibroblasts, in particular, are widely reported to play roles in wound repair. In our study, FIB_2 exhibited an inflammatory phenotype, secreting CXCL5 and CXCL6 to interact with epithelial cells via CXCR2. Additionally, FIB_2 expressed ageing‐associated genes, including *POSTN* and *SPARC*, which are key components of the SASP [56]. Interestingly, FIB_2 also displayed molecular signatures characteristic of myofibroblasts, known to produce inflammatory mediators that influence the tissue microenvironment. The senescence‐associated inflammatory phenotype and myofibroblast‐like features of FIB_2 may exacerbate epithelial inflammation and impair re‐epithelialization. In turn, epithelial cells contribute to this crosstalk by secreting cytokines that target fibroblasts, facilitating their differentiation and promoting submucosal fibrosis. For instance, in our study, epithelial cells secreted pro‐fibrotic factors such as TGFB1 and TGFA, which likely act on FIB_2 to perpetuate this pathological process. In summary, these findings provide a mechanistic explanation for the observed inflammatory phenotypes and elucidate the epithelial–stromal interaction in post‐ESD wound healing.

Our study has several limitations. Firstly, the sample size was relatively small, emphasising the need for additional studies to confirm our findings. Second, although we identified and experimentally validated an increased abundance of the specific subpopulations of epithelial cells and fibroblasts, the heterogeneity of other cell populations and functional phenotypic assays were not conducted. Furthermore, although we observed differences in ligand–receptor pairs between fibroblasts and epithelial cells, further validation of gene expression and downstream signalling pathways is required.

## 5 Conclusion

In summary, we compiled the single‐cell transcriptome profiles of normal and stricture oesophageal tissues post‐ESD, uncovering heterogeneity in epithelial cells and fibroblasts. We identified a subcluster of FTH1^hi^ DFSC_2 associated with hyperkeratosis and noted myofibroblast activation and epithelial–stromal interactions in stricture tissues. These findings shed light on the mechanisms of oesophageal stricture formation, with potential translational relevance for improving patient outcomes.

## Author Contributions

**Lulong Tao:** investigation (equal), methodology (equal), software (equal), writing – original draft (equal). **Junjun Xia:** methodology (equal), validation (equal). **Die Hu:** software (equal), visualization (equal). **Guoxin Zhang:** project administration (equal), writing – review and editing (equal). **Yaoyao Gong:** project administration (equal), writing – review and editing (equal). **Jin Yan:** funding acquisition (equal), project administration (equal), writing – review and editing (equal).

## Ethics Statement

This study was approved by the Ethics Committee of the First Affiliated Hospital of Nanjing Medical University. The experiments concerning animals were approved by the Nanjing Medical University Ethics Committee.

## Consent

Written informed consent forms were obtained from all recruited patients.

## Conflicts of Interest

The authors declare no conflicts of interest.

## Supporting information


Tables S1–S5.

Figures S1–S9.

## Data Availability

The data that support the findings of this study are available from the corresponding authors upon reasonable request.
