# Supplementary material for: Single‐Cell Sequencing Reveals Heterogeneity and Interactions Between Epithelial Cells and Fibroblasts in Post‐ESD Oesophageal Stricture
Source: J Cell Mol Med. 2025 Feb 5;29(3):e70411. doi: 10.1111/jcmm.70411 (PMC11798872; doi:10.1111/jcmm.70411)
Supplement: Supplementary file 2 — Figures S1–S9. [file JCMM-29-e70411-s002.pdf]

Supplementary materials

Single-cell sequencing reveals heterogeneity and interactions between epithelial cells and fibroblasts in post-ESD esophageal stricture

Lulong Tao[1,2], Junjun Xia[1,2], Die Hu[1,2], Guoxin Zhang[1,2],Yaoyao Gong[1,2] and Jin Yan[1,2]

[1]Department of Gastroenterology, The First Affiliated Hospital with Nanjing Medical University

[2]The First Clinical Medical College, Nanjing Medical University

Lulong Tao and Junjun Xia contributed equally to this work.

Correspondence:

Jin Yan, Department of Gastroenterology, The First Affiliated Hospital with Nanjing Medical University, 300 Guangzhou Road, Gulou District, Nanjing, Jiangsu 210000, China. E-mail: yanjinjsph@njmu.edu.cn

Yaoyao Gong, Department of Gastroenterology, The First Affiliated Hospital with Nanjing Medical University, 300 Guangzhou Road, Gulou District, Nanjing, Jiangsu 210000, China. E-mail: ygong@njmu.edu.cn

Guoxin Zhang, Department of Gastroenterology, The First Affiliated Hospital with Nanjing Medical University, 300 Guangzhou Road, Gulou District, Nanjing, Jiangsu 210000, China. E-mail: guoxinz@njmu.edu.cn

Workflow of the analysis

Primary analysis of raw read data (scRNA-seq)

Raw reads were processed to generate gene expression profiles using CeleScope v1.15.0 (Singleron Biotechnologies) with default parameters. Briefly, Barcodes and UMIs were extracted from R1 reads and corrected. Adapter sequences and poly A tails were trimmed from R2 reads and the trimmed R2 reads were aligned against the GRCh38 (hg38) transcriptome using STAR(v2.6.1b). Uniquely mapped reads were then assigned to genes with FeatureCounts(v2.0.1). Successfully Assigned Reads with the same cell barcode, UMI and gene were grouped together to generate the gene expression matrix for further analysis.

Quality control, dimension-reduction and clustering (Scanpy)

Scanpy v1.8.1 was used for quality control, dimensionality reduction and clustering under Python 3.7. For each sample dataset, we filtered expression matrix by the following criteria: 1) cells with gene count less than 200 or with top 2% gene count were excluded; 2) cells with top 2% UMI count were excluded; 3) cells with mitochondrial content > 50% were excluded; 4) genes expressed in less than 5 cells were excluded. After filtering, 40906 cells were retained for the downstream analyses, with on average 1161.728 genes and 5236.286 UMIs per cell. The raw count matrix was normalized by total counts per cell and logarithmically transformed into normalized data matrix. Top 2000 variable genes were selected by setting flavor = 'seurat'. Principle Component Analysis (PCA) was performed on the scaled variable gene matrix, and top 19 principle components were used for clustering and dimensional reduction. Cells were separated into 23 clusters by using Louvain algorithm and setting resolution parameter at 1.2. Cell clusters were visualized by using Uniform Manifold Approximation and Projection (UMAP)

Wolf, F., Angerer, P. & Theis, F. SCANPY: large-scale single-cell gene expression data analysis. Genome Biol 19, 15 (2018). https://doi.org/10.1186/s13059-017-1382-0

Batch Effect removal

Harmony: Batch effect between samples was removed by Harmony v1.0 using the top 20 principal components from PCA.

Butler, A., Hoffman, P., Smibert, P. et al. Integrating single-cell transcriptomic data across different conditions, technologies, and species. Nat Biotechnol 36, 411–420 (2018). https://doi.org/10.1038/nbt.4096

Differentially expressed genes (DEGs) analysis (scanpy)

To identify differentially expressed genes (DEGs), we used the scanpy.tl.rank_genes_groups() function based on Wilcoxon rank sum test with default parameters, and selected the genes expressed in more than 10% of the cells in either of the compared groups of cells and with an average log(Fold Change) value greater than 0.25 as DEGs. Adjusted p value was calculated by benjamini-hochberg correction and the value 0.05 was used as the criterion to evaluate the statistical significance.

Pathway enrichment analysis

To investigate the potential functions of cell clusters, Gene Ontology (GO) and Kyoto Encyclopedia of Genes and Genomes (KEGG) analysis were used with the "clusterProfiler" R package v 4.0.0. Pathways with p_adj value less than 0.05 were considered as significantly enriched. Selected significant pathways were plotted as bar plots.

Celltype annotation

Cell-type recognition with Cell-ID

Cell-ID is multivariate approach that extracts gene signatures for each individual cell and perform cell identity recognition using hypergeometric tests(HGT). Dimensionality reduction was performed on normalized gene expression matrix through multiple correspondence analysis, where both cells and genes were projected in the same low dimensional space. Then a gene ranking was calculated for each cell to obtain most featured gene sets of that cell. HGT were performed on these gene sets against Esophagus reference from SynEcoSys database, which contains featured genes of all cell-type in the specific organ/tissue. Identity of each cell was determined as the cell-type has the minimal HGT p value. For cluster annotation, Frequency of each cell-type was calculated in each cluster, and cell-type with highest frequency was chosen as the identity of the cluster.

Reference:

1. Cortal, A., Martignetti, L., Six, E. & Rausell, A. Gene signature extraction and cell identity recognition at the single-cell level with Cell-ID. Nature Biotechnology 1–8 (2021) doi:10.1038/s41587-021-00896-6.
2. Yan Zhang, Bingyu Li, Jiachen Duan, Xuezhen Chen, Xiaogang Zhang, Jun Ye, Ana Veloso, Jue Fan, Nan Fang. Preprint at bioRxiv https://doi.org/10.1101/2023.02.14.528566

The cell type identification of each cluster was determined according to the expression of canonical markers from the reference database SynEcoSys™ (Singleron Biotechnology). SynEcoSys™ contains collections of canonical cell type markers for single-cell seq data, from CellMakerDB, PanglaoDB and recently published literatures.

Subtyping of major cell types

To obtain a high-resolution map of Epithelial Cells, Differentiating Suprabasal Cells, Fibroblasts, cells from the specific cluster were extracted and reclustered for more detailed analysis following the same procedures described above and by setting the clustering resolution as 1.2, 0.3,0.3.

Yan Zhang, Bingyu Li, Jiachen Duan, Xuezhen Chen, Xiaogang Zhang, Jun Ye, Ana Veloso, Jue Fan, Nan Fang. Preprint at bioRxiv https://doi.org/10.1101/2023.02.14.528566

Filtering Cell Doublets

Cell doublets were estimated based on the expression pattern of canonical cell markers. Any clusters enriched with multiple cell type-specific markers were excluded for downstream analysis.

Cell-cell Interaction Analysis (CellChat)

CellChat (version 1.6.1) was used to analyze the intercellular communication networks from scRNA-seq data. A CellChat object was created using the R package process. Cell information was added into the meta slot of the object. The ligand-receptor interaction database was set, and the matching receptor inference calculation was performed.

Jin S, Guerrero-Juarez C F, Zhang L, et al. Inference and analysis of cell-cell communication using CellChat. Nature communications, 2021, 12(1): 1-20.

Cell Differentiation Potential Evaluation: CytoTRACE

CytoTRACE v 0.3.3 (a computational method that predicts the differentiation state of cells from single-cell RNA-sequencing data using gene counts and expression) was used to predict the differentiation potential of cell subpopulations.

Gulati G S, Sikandar S S, Wesche D J, et al. Single-cell transcriptional diversity is a hallmark of developmental potential. Science, 2020, 367(6476): 405-411.

Pseudotime Trajectory Analysis: monocle2

Cell differentiation trajectory of monocyte subtypes was reconstructed with the Monocle2 v 2.22.0. For constructing the trajectory, top 2000 highly variable genes were selected by Seurat(v 4.1.0) FindVairableFeatures(), and dimension-reduction was performed by DDRTree(). The trajectory was visualized by plot_cell_trajectory() function in Monocle2.

Xiaojie Qiu, Andrew Hill, Cole Trapnell et al (2017): Single-cell mRNA quantification and differential analysis with Census. Nature Methods.

AUCell Geneset Enrichment Analysis

To investigate the keratinization activity in DFSC_2, “GOBP_KERATINIZATION” pathways were collected and used as functional gene sets for AUCell scoring. AUCell scores of gene sets were visualized by using FeaturePlot()/VlnPlot() in Seurat. P-values from t test were used for estimating the statistical significance between cell types and groups.

Aibar S, Gonzalez-Blas CB, Moerman T, Huynh-Thu VA, Imrichova H,Hulselmans G, et al. SCENIC: single-cell regulatory network inference and clustering. Nat Methods 2017;14:1083–6..

Transcription factor regulatory network analysis (pySCENIC)

Transcription factor network was constructed by pyscenic (v0.11.0) using scRNA expression matrix and transcription factors in AnimalTFDB. First, GRNBoost2 predicted a regulatory network based on the co-expression of regulators and targets. CisTarget was then applied to exclude indirect targets and to search transcription factor binding motifs. After that, AUCell was used for regulon activity

quantification for every cell. Cluster-specific TF regulons were identified according to Regulon Specificity Scores (RSS) and the activity of these TF regulons were visualized in heatmaps.

Van de Sande, Bram, et al. "A scalable SCENIC workflow for single-cell gene regulatory network analysis." *Nature Protocols* 15.7 (2020): 2247-2276.

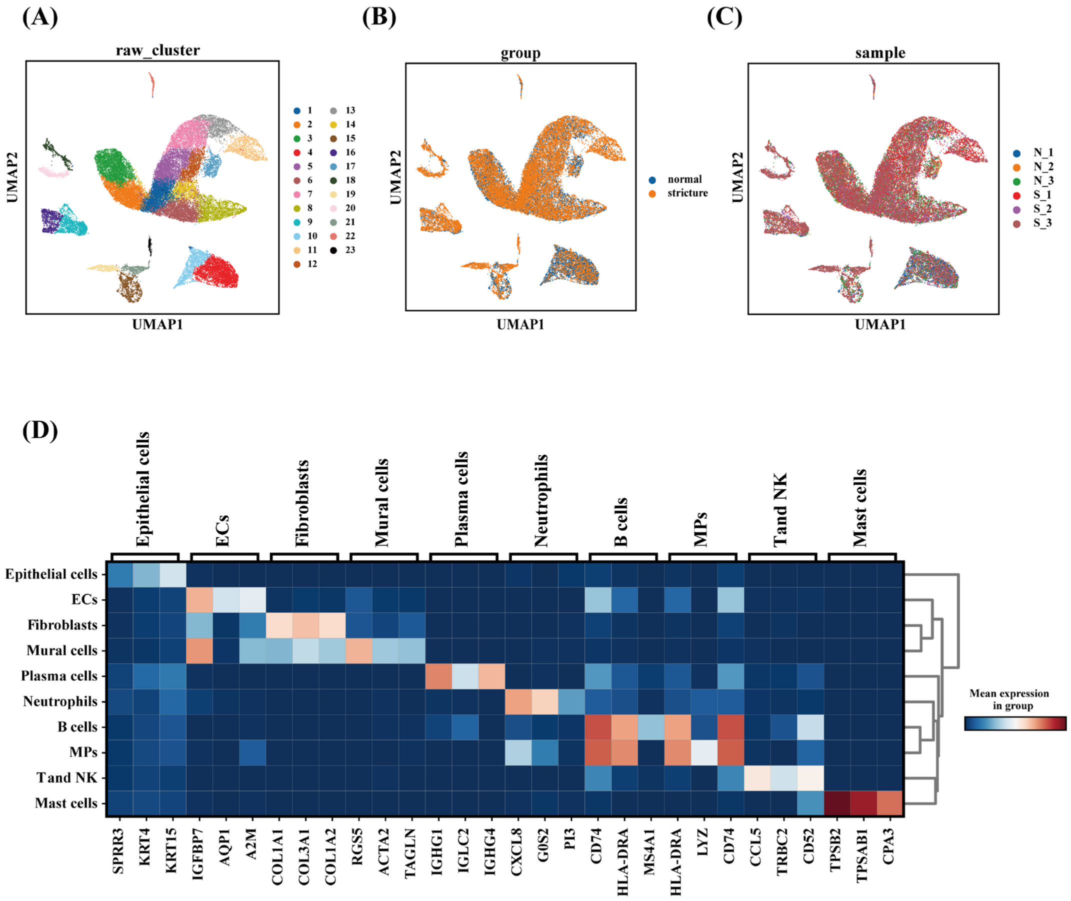

Figure.S1: Batch Integration of Data and heatmap of differentially expressed genes.(A)UMAP plot of integration of 23 raw clusters.((B)UMAP plot of all cells at resolution 1.2 divided by group.(C)UMAP plot of all cells at resolution 1.2 divided by samples.(D) Heatmap showing the respective differentially expressed genes of the major cell types.

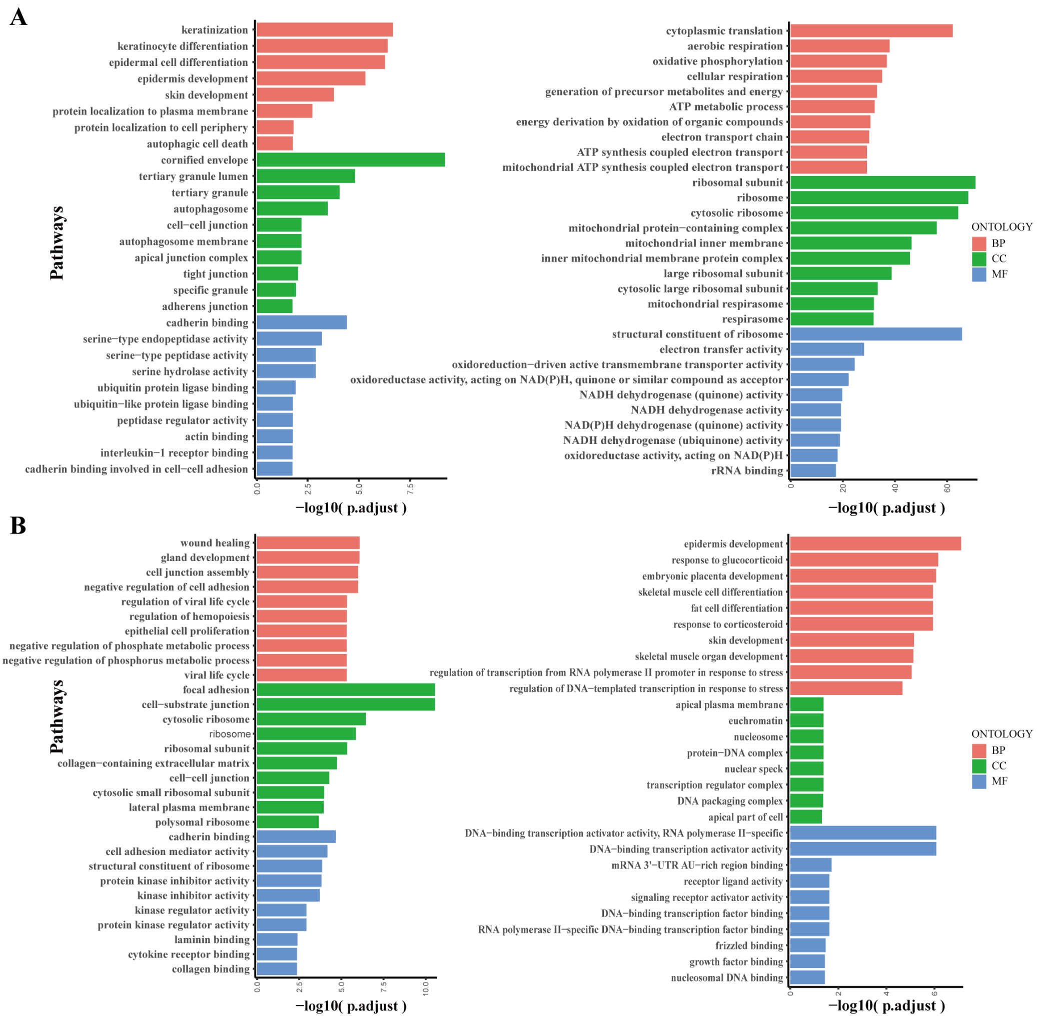

Figure.S2:GO analysis of the DEGs between normal and stricture tissues in QBCs and DFSC_2.(A)Barplot showing the Gene Ontology Analysis results of the up- (left)and down-regulated(right) differentially expressed genes between groups in DFSC_2.(B) Barplot showing the Gene Ontology Analysis results of the up- (left)and down-regulated(right) differentially expressed genes between groups in QBCs. BP:Biological Process ;CC:Cellular Component ;MF:Molecular Function.

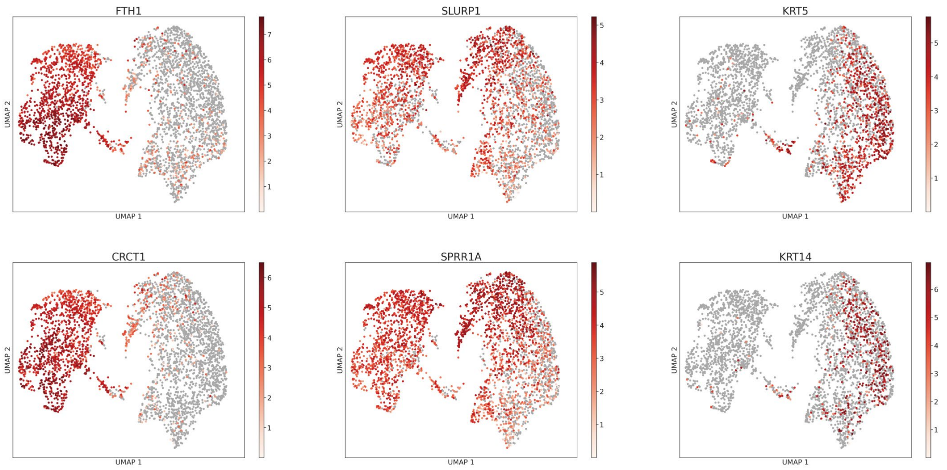

Figure.S3: Featureplots showing the distribution of top differential gene in subclusters of DFSC_2

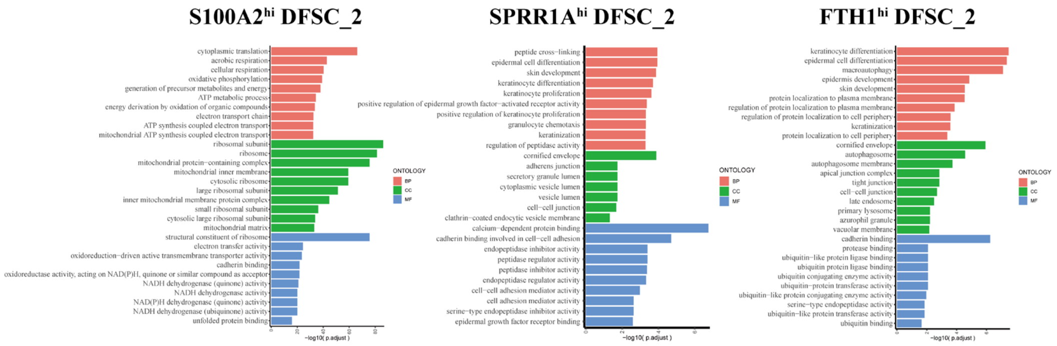

Figure.S4: Functional enrichment analysis of the three subclusters in DFSC_2.

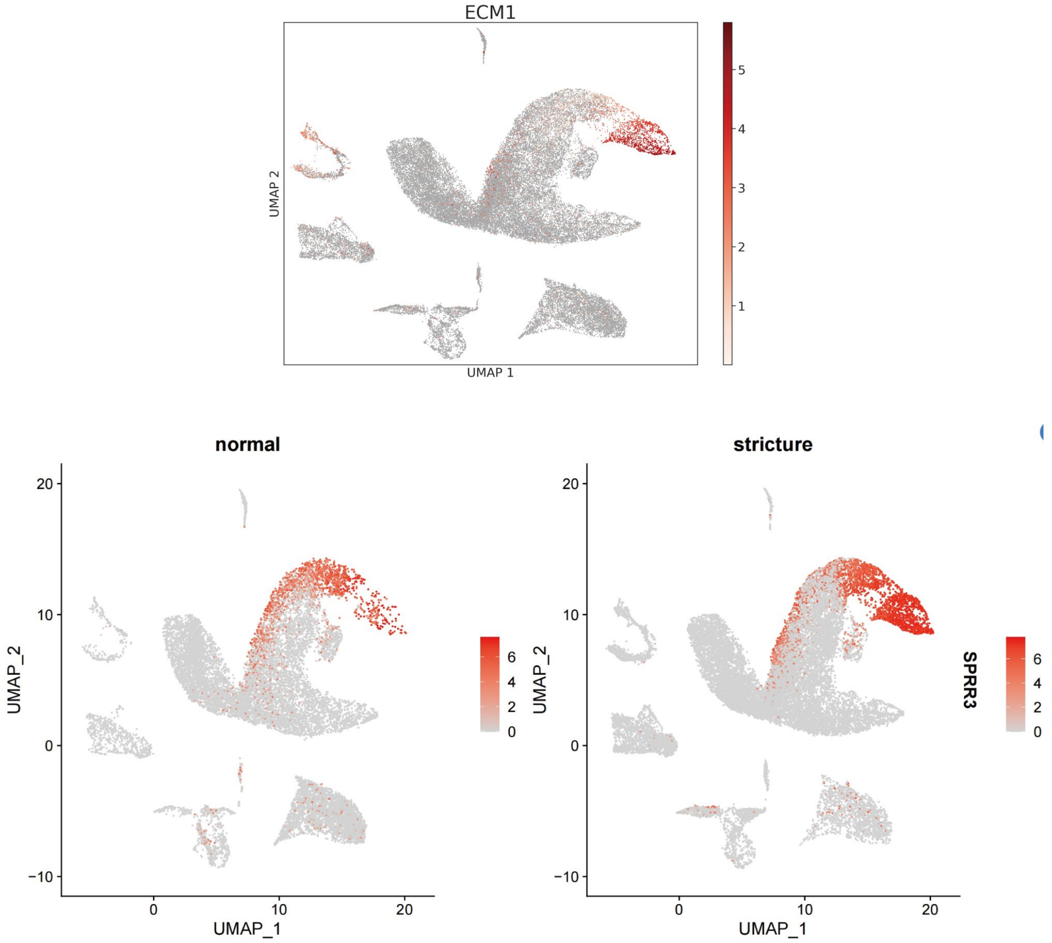

Figure.S5:Feature plots demonstrating the distribution of ECM1 and SPRR3 gene expression.

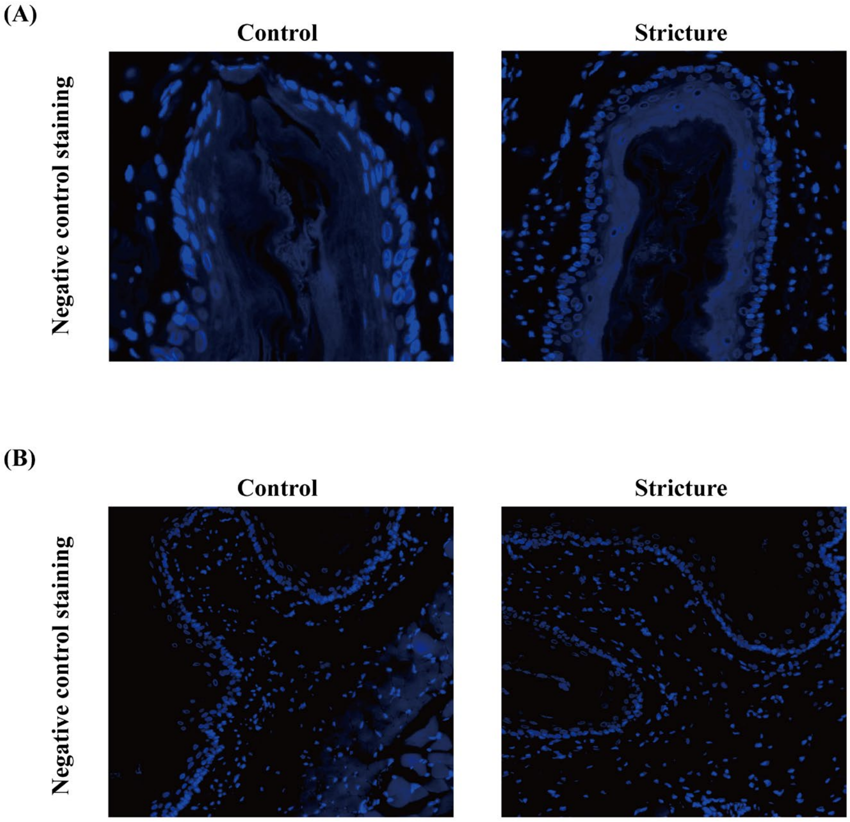

Figure.S6: Negative control staining of immunofluorescence.(A)Negative control staining of SPRR3 and ECM1 of the immunofluorescence validation of the FTH1^{hi} DFSC_2.(B) Negative control staining of CTHRC1 and POSTN of the immunofluorescence validation of the FIB_2.

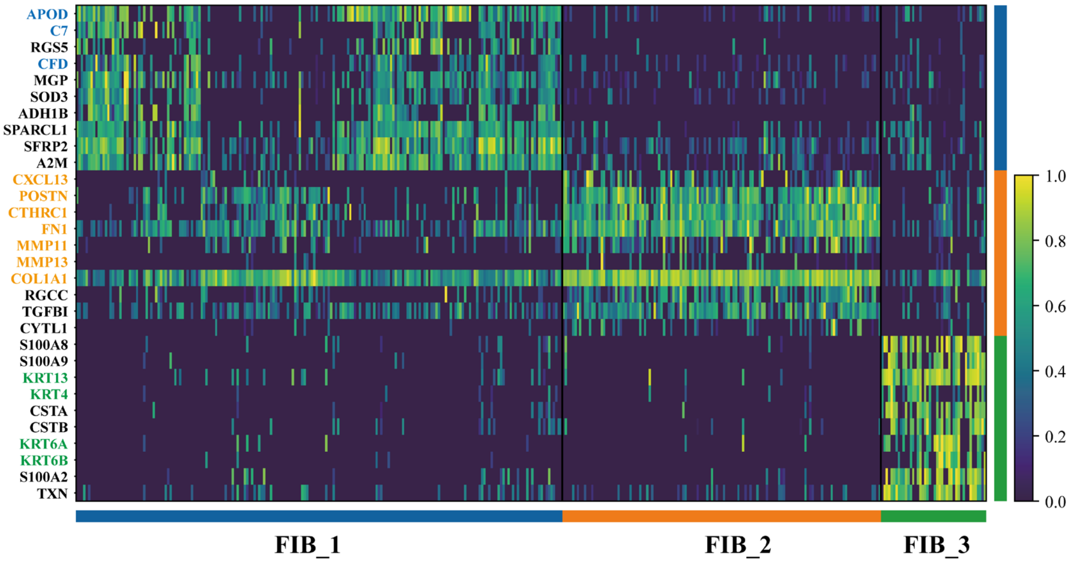

Figure.S7: Heatmaps of differential gene expression in each subpopulation of fibroblasts.

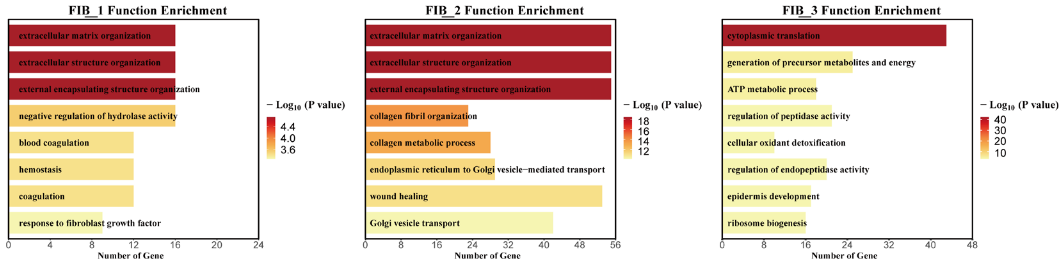

Figure.S8: GO functional enrichment for the three subclusters of fibroblasts.

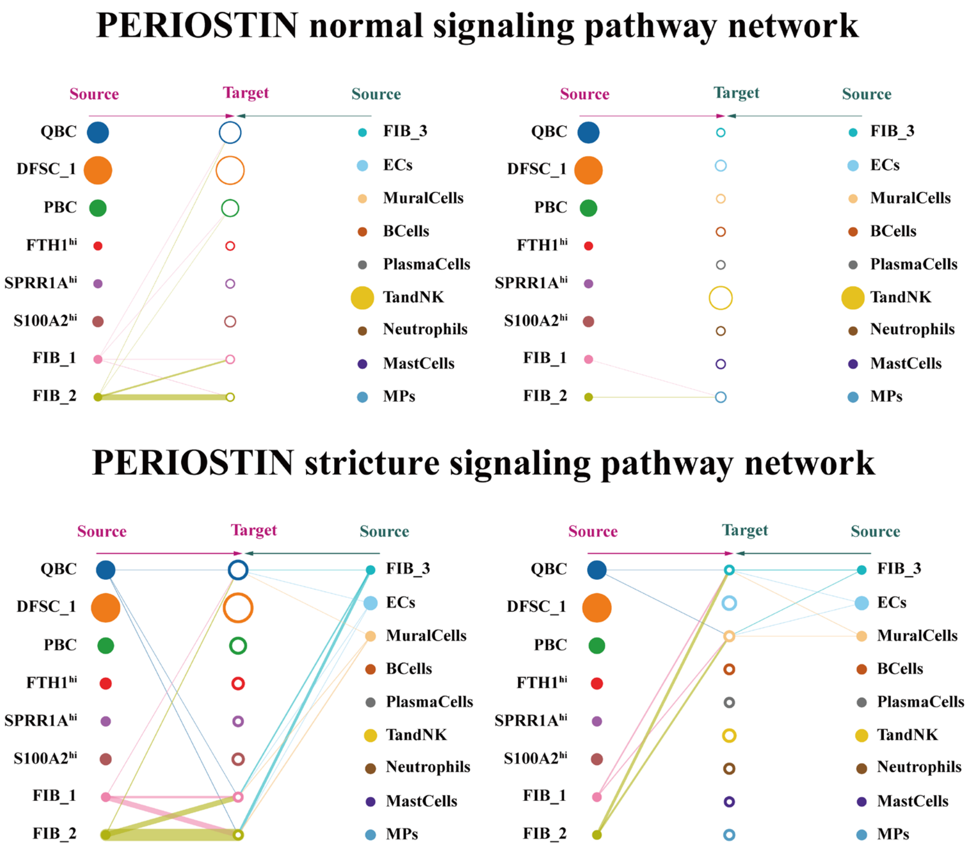

Figure.S9: Hierarchy plot showing the source and target change of the PERIOSTIN signaling pathway between groups.
